# Association between acute critical life events and the speed of onset of depressive episodes in male and female depressed patients

**DOI:** 10.1186/s12888-018-1923-4

**Published:** 2018-10-16

**Authors:** Maria Strauss, Roland Mergl, Nora Gürke, Kerstin Kleinert, Christian Sander, Ulrich Hegerl

**Affiliations:** Department of Psychiatry and Psychotherapy, University of Leipzig, Semmelweisstr. 10, 04103 Leipzig, Germany

**Keywords:** Major depression, Beginning, Unipolar depression, Gender differences, Speed of onset

## Abstract

**Background:**

A series of studies indicate that a fast onset of a depressive episode (within 7 days) is a clinical variable useful for indicating bipolarity even when no manic episode has occurred to date. The role of acute critical life events as an external trigger for a fast onset of the depression is unclear so far. Therefore, aim of this investigation was to analyse the effects of acute critical life events on the speed of onset of depressive episodes.

**Methods:**

Speed of onset of depression was assessed using the patient interview “Onset of Depression Inventory”. Acute critical life events occurring within the last 6 months before the onset of first depressive symptoms were assessed using the Munich Interview for the Assessment of Life Events and Conditions.

**Results:**

96 of 100 (96.0%) patients had at least one acute critical life event within six months prior to first symptoms of a depressive episode. 22 patients (22.0%) had a fast onset of depression (≤ 7 days). Faster onset of the current depressive episode was significantly associated with a higher number of acute minor life events (β = − 0.23; *p* = 0.02), but overall fast onset of a depressive episode was not significantly associated with more acute critical life events in the six months before the onset of the depression. The association between the number of acute critical life events in the half-year period preceding the onset of unipolar depressive disorders and speed of onset for the current depressive episode was neither dependent from gender nor the presence of prior depressive episodes.

**Conclusions:**

Speed of onset of depression is not strongly influenced by external trigger e.g. acute critical life events.

**Electronic supplementary material:**

The online version of this article (10.1186/s12888-018-1923-4) contains supplementary material, which is available to authorized users.

## Background

Stressful life events often precede the onset of depression [1] and this relationship appears to be more pronounced in women than in men. So far, the causes for these gender differences are still unclear [2, 3].

Brown and Harris [4] determined that life events that occur in the 6 months prior to depression onset in contrast to life events going further back in time than 6 months are the most central in precipitating that onset. Further there is a consistent evidence for a dose-response relationship between stressful events and depression, with severe events more strongly associated with depression than non-severe events [1]. However, there are also indications that certain depressive subtypes (e.g. melancholic subtype) show a different vulnerability to critical live events [5, 6]. In this context it is important to note that individuals have been shown to be more sensitive to the effects of minor life events after having a depressive episode such that minor events in the sense of life events not exceeding a mildly stressing level predict recurrence of depression for medicated patients over 3 years [7] and are more likely to predict recurrence of depression than the first onset of a depressive episode [8].

In summary, the role of critical life events in the development of a depression seems dependent on multiple factors and has not yet been conclusively clarified. This supports the approach that further investigations on the link between critical life events and the onset of depression must also consider subtle differences in the onset, development and clinical presentation of depressive episodes. One possible important aspect in this process is to investigate the association between clinical life events and the speed of onset of a depressive episode.

While some studies have examined the relationship between stressful life events that occurred before the onset of the depressive episode and speed of recovery of these episodes [9], a systematic investigation on the influence of acute life events on the speed of onset of depression has not been performed so far.

The development of depressive episodes from first symptoms to full-blown symptomatology can be very rapid, taking less than 1 hour in some patients, or very slow, taking up to months. A number of studies [10, 11] indicate that the speed of onset of a depressive episode is a clinical characteristic useful for indicating a hidden bipolarity even when no manic episode has occurred so far. Furthermore, these studies observed in patients with recurrent depressive episode a significant association between the speed of onset of the current and the previous depressive episode, indicating that this clinical characteristic has an intra-individual stability and is possibly useful for identifying more homogenous subgroups with stronger genetic diathesis for depression in depressive patients. In this context, it could be assumed that individuals with a genetic vulnerability regarding depression are more frequently found in the subgroup of patients with a fast onset of depressive episodes. It can be hypothesized that protective epigenetic mechanisms are weak in this group. Thus, it can be expected that stress can smoothly damage these mechanisms, induce maladaptive neuronal modification and have a share in fast onset of depressive episodes via this neurobiological pathway [12].

The “Onset of Depression Inventory” (ODI) is a structured patient interview designed to measure the speed of onset of a depressive episode, that is, the period from the first depressive symptom to a fully blown syndrome (for more details see [10, 11]). Previous studies have shown a good test-retest stability of the ODI with a reliability of rho = 0.83 [13]. In the previous ODI-studies all patients were not included who had experienced an acute life event in the last 2 weeks before the onset of the current depressive episode, in order to eliminate the chance of such an event triggering a sudden onset of depression [10, 11]. However, to date it has remained unclear how the experience of critical life events triggers an abrupt beginning of the depression.

The assumption that acute critical life events can trigger fast onset of depression is obvious but does not mean that patients with a fast onset of depression are characterized by an increased vulnerability regarding critical life events as patients with “hidden” bipolarity seem to be characterized by both lack of acute critical life events preceding their depressive episode and a fast onset of depression. The matter is quite complicated: People with unipolar depression may have fast onsets when a severe life event occurs, and bipolar depressed patients may also have a fast onset. Furthermore, there is some indication that major life events in the sense of at least markedly stressing life events might also trigger bipolar depression, so the possible predictions become even more complicated (i.e., the genetic liability for bipolar disorder may be due to sensitivity to stress, or due to biogenetic factors independent of stress). In view of these issues we decided to focus on patients with unipolar depression in the following. In this context, it will be important to characterize critical life events experienced prior the onset of the depression regarding their number, type, unpleasantness and burden in a systematic way to determine predictors for a relationship between a fast onset of the depression and critical life events. Thus, it will be of special interest to assess different types of life events like major, minor, foreseeable, unforeseeable, positive, negative, and neutral life events; the assessment of their mean burden and unpleasantness will be important, too.

Against this background, the aim of this study was to investigate the effects of acute critical life events on the speed of onset of the depressive episode in patients with a current unipolar depression with respect to gender differences by using standardized instruments. For this purpose, we have investigated an entirely new sample without any overlap with other samples of patients with depression assessed by using the ODI whose data had been published by now (e.g., [10. 11, 13]). In view of studies suggesting that the prevalence of major depression is higher in women than in men partly due to biological factors like a disruption of the estrous cycle (for review see [14]) and women are more often exposed to negative life events than men [3] it can be expected that negative life events trigger a fast onset of depressive episodes more often in women than in men.

Within an exploratory approach, we investigated whether having a first onset or prior episode of depression had a significant influence on the association between critical life events and speed of onset of the current depressive episode.

## Methods

### Subjects

Within the context of an open, cross-sectional clinical observational study 152 consecutive inpatients suffering from a depressive episode (2 Day clinic patients) had been recruited in the Department of Psychiatry and Psychotherapy (University Hospital of Leipzig, Germany). Data was collected between May 2013 and August 2015.

Main inclusion criteria were as follows: minimum age of 18 years; ability of the patient to communicate with others and to fulfil the demands of the study; written informed consent according to the guidelines laid down in the current version of the Declaration of Helsinki; clinical diagnosis of depressive episodes (according to the diagnostic criteria of the “International Classification of Diseases” (tenth edition) [15] (ICD-10): F32.0, F32.1, F32.2, F32.8); recurrent depressive disorders (ICD-10: F33.0, F33.1, F33.2, F33.8). A combination of depressive episodes or recurrent depressive disorders with cyclothymia (according to ICD-10: F34.0) or dysthymia (according to ICD-10: F34.1) was in accordance with the study protocol.

Patients had to be excluded if they fulfilled the diagnostic criteria of the following disorders: Bipolar affective disorders, dysthymia, other persistent depressive disorders without depressive episodes like cyclothymia, addiction or abuse regarding alcohol, hypnotics or illicit drugs, psychotic disorders, dementia, adjustment disorder, comorbid borderline personality disorder, and severe acute somatic diseases requiring treatment (especially life-threatening diseases like cancer). Further exclusion criteria were: pregnancy, duration of the current depressive episode being more than 2 years, acute suicidality (as assessed in the context of the clinical examinations) and a strong association between the onset of the current depressive episode, change of psychopharmacological treatment strategies within 14 days before the onset of the current depressive episode and inconsistent findings regarding the speed of onset of the current depressive episode.

Clinical diagnoses were made by physicians with clinical expertise and based on the diagnostic criteria from the ICD-10 [15]. The physicians who were involved in the study had at least 3 years of clinical experience in diagnostics.

### Clinical instruments

The speed of onset of current and preceding depressive episodes was investigated by using the “Onset of Depression Inventory” (ODI). Details regarding this inventory which had been conceptualized for patients with depressive episodes can be found elsewhere [10, 11, 13].

In brief, the patients gave a free verbal assessment on how long it took for their current depressive episode to fully develop in the context of the ODI interview. This information was then converted to days.

The Munich Interview for the Assessment of Life Events and Conditions (MEL) [16] was selected as a standardized instrument for the assessment of different types of critical life events occurring within 6 months before the onset of first symptoms of a current depressive episode.

The MEL encompasses 85 items (including positive and negative, major as well as minor life events and life situations which can be related to eleven social areas: education (e.g., failing an examination), job and household (e.g., cancellation), sexual relationships (e.g., submission of divorce), pregnancy/children (e.g., aborts), family (e.g., curving out from the parental home), social contacts (e.g., termination of friendships), cases of death (e.g., spouse), habitation (e.g., relocation), financial events (e.g., commercial breakdown), violation of law (e.g., prison sentence) and illness/health (e.g., hospital stay)). 27 items refer to chronic life events (items 16–21,25,32,33,36-39,48,49,52,53,55,59,62,63,71,75,78,82–84), the other 58 items to acute life events. The latter items relate to a period of 6 months before the onset of the current depressive episode; corresponding events can occur more than once in this period and are separately coded. The burden by each life event was rated by using a 5 point scale (1: not stressing; 2: mildly stressing; 3: markedly stressing; 4: strongly stressing; 5: extremely stressing). In a similar way, it had been assessed whether a certain life event was considered to be positive or negative (by using a 5-point scale regarding unpleasantness with the categories 1: very positive; 2: positive; 3: neither positive nor negative (neutral); 4: negative; 5: very negative). Regarding foreseeability of life events, they could be coded as either foreseeable in the long term (> 3 months) (score 1), short term foreseeable (less than 3 months) (score 2) (with foreseeable life events being scored 1 or 2) or unforeseeable (less than 1 week). For each MEL item the following variables had been computed: number of life events in the preselected period; number of foreseeable life events; number of unforeseeable life events; number of positive life events (unpleasantness: 1 or 2); number of neutral life events (unpleasantness: 3); number of negative life events (unpleasantness: 4 or 5); mean patient-specific burden by the life events belonging to the selected MEL item; mean patient-specific evaluation of the unpleasantness elicited by the life events belonging to the selected MEL item; number of minor life events (e.g.: “You have married.” (event assessed as mildly stressing); burden by the life event: 1 or 2); number of major life events (e.g.: “Your child has died.” (event assessed as extremely stressing); burden by the life event: at least 3). Based on this data, the following variables could be computed for each patient: Number of all (acute as well as chronic)/only acute critical life events preceding the onset of the current depressive episode; corresponding frequencies for foreseeable and unforeseeable acute life events; corresponding frequencies for positive, neutral and negative acute life events; corresponding frequencies for major and minor acute life events; mean burden by acute critical life events; mean unpleasantness elicited by acute critical life events. The reliability of the MEL is well established [16].

The following clinical data had been additionally collected: age at the onset of the depressive disorder, number of suicide attempts and the total score of the Hamilton Depression Rating Scale (HDRS) [17].

The clinical examinations were conducted by two raters. The two raters got information about the dates of the life events.

### Statistical analysis

The analyses were performed for the MEL variables regarding acute critical life events as well as the speed of onset of the current depressive episode (in days).

Normal distribution of the main variables was tested by the Kolmogorov-Smirnov test. Gender differences regarding MEL variables and speed of onset of the current depressive episode were tested for statistical significance by using the t-test for independent sample comparison (in case of normal distribution of the dependent variable) or the non-parametric Mann-Whitney U test (if the Kolmogorov-Smirnov test provided a *p* value < 0.05 or the group size was low (< 15)). Analogously, patients with depressive episodes and patients with recurrent depressive disorders had been compared.

Pearson’s correlation coefficients and Spearman-Brown correlation coefficients (in case of non-normally distributed or rank-scaled variables) were computed in order to explore the association between number, mean burden and unpleasantness of acute critical life events and speed of onset of the current depressive episode. These coefficients were computed for the total sample. Moreover, the relationship between age, the sum score of the HDRS and further clinical variables (regarding critical life events and speed of onset of the current depressive episode) was investigated in an analogous way. If age and/or the sum score of the HDRS were found to be significantly associated with both MEL variables and speed of onset of the current depressive episode, partial correlation coefficients were chosen to examine the association between number, mean burden and unpleasantness of acute critical life events and speed of onset of the current depressive episode by using age and/or severity of depression as covariates. If age and/or the sum score of the HDRS were only significantly associated with one of the afore-mentioned variables semi-partial correlation coefficients were selected.

In addition, we conducted a linear regression analysis (by using the backward elimination method; threshold: *p* < 0.10) with the criterion being the speed of onset of the current depressive episode (in days) and the predictors being variables reflecting acute critical life events in order to be able to show which critical life event variables had the most pronounced impact on the speed of onset of depression.

Regression coefficient estimates (β) with corresponding 95% confidence intervals (CI) and *p* values were computed; the same was true for the proportion of variance accounted for by the regression model as a whole (R^2^).

In this context, inter-correlations between the predictors had been investigated by using Spearman-Brown correlation coefficients (rho) in order to assess their multicollinearity; rho ≥0.70 had been selected as critical threshold in this context. The corresponding findings are summarized in the appendix (see Additional file 1: Table S1).

17 of 55 correlation coefficients (30.91%) were found to be ≥0.70. Thus we decided to reduce the number of variables by conducting a principal component factor analysis (PCA) with subsequent Varimax rotation on them. For this purpose, the correlation coefficients for each pair of z-standardized variables had been calculated. The Scree test was used for the factor extraction and the variables with the highest loadings on one factor combined with low loadings on the other factors were identified. The corresponding scores were selected as independent variables for the subsequent regression analysis.

The results of the factor analysis can be found in Table 1.

**Table 1:** Rotated component matrix for the factor analysis of scores of 100 patients with unipolar depressive disorders on different variables reflecting acute critical life events derived from the Munich Interview for the Assessment of Life Events and Conditions (MEL)

Variables	Component 1	Component 2	Component 3
Total number of critical life events	0.876	0.251	0.023
Total number of acute critical life events	0.890	0.431	0.123
Total number of unforeseeable acute critical life events	0.771	0.099	0.107
Total number of foreseeable acute critical life events	0.537	0.532	0.074
Total number of acute positive critical life events	0.420	0.842	−0.163
Total number of acute neutral critical life events	0.114	0.108	0.981
Total number of acute negative critical life events	0.940	−0.146	−0.030
Total number of acute minor critical life events	0.437	0.800	0.155
Total number of acute major critical life events	0.942	−0.050	0.049
Mean unpleasantness of acute critical life events	0.166	−0.917	−0.033
Mean burden of acute critical life events	0.089	−0.877	−0.168

Notes: Extraction method: Principal component factor analysis (PCA); rotation method: Varimax

The PCA resulted in three factors (eigenvalues: 1.01–5.52) explaining 84.02% of the total variance. Based on the factor loadings, the three factors can be interpreted as reflecting (1) major and negative, (2) minor and positive and (3) neutral acute critical life events. Thus, the following three variables remained as predictors: the frequencies for acute major, neutral and minor critical life events.

To test our hypothesis that the association between critical life events and the speed of onset for the current depressive episode is moderated by gender we applied multiple hierarchical linear regression models with the speed of onset of the current depressive episode representing the criterion variable and type of critical life events (as resulting from the results of the afore mentioned PCA), gender, and the interaction effects between type of critical life events and gender being the predictor variables. In this context, the product of the last-mentioned variables was entered into the regression equation after inputting the two main effects [18]. The gender hypothesis was confirmed if the interaction effect between type of critical life events and gender on speed of onset for the current depressive episode proved to be statistically significant.

In an analogous way, it was investigated whether the association between critical life events and the speed of onset for the current depressive episode was moderated by the presence of prior depressive episodes.

The SPSS version 20.0 was utilized for the afore-mentioned statistical analyses and the significance level was set at α = 0.05. A Bonferroni correction for multiple comparisons had been applied, if necessary. All statistical tests were two-sided.

## Results

### Demographic and clinical characteristics

152 patients had been recruited. 26 patients (17.11%) had to be excluded because of inadequate diagnoses: In 11 cases, a bipolar affective disorder (ICD-10: F31) had been the main diagnosis, in 9 cases a depressive episode had been associated with mental and behavioral disorders due to psychoactive substance use (ICD-10: F10-F19) (with one case additionally fulfilling the diagnostic criteria of a borderline personality disorder). Further not fitting diagnoses were as follows: severe depressive episode with psychotic symptoms (ICD-10: F32.3) (*n* = 2); adjustment disorder (ICD-10: F43.2) (*n* = 1); comorbid emotionally unstable personality disorder, borderline type without further additional psychiatric diagnoses (ICD-10: F60.31) (*n* = 3). In further 11 cases (7.24%) there had been changes of psychotropic medication within the last 14 days before the onset of the current depressive episode leading to an exclusion from the present study (in two cases combined with inadequate diagnoses). Moreover, five other patients (3.29%) had to be excluded because the duration of their current depressive episode had been longer than 2 years. In addition, ten cases (6.58%) were characterized by divergent ODI and MEL data regarding the speed of onset of the current depressive episode. Thus, the final sample consisted of 100 persons (65.79% of the original sample).

61 patients were females. The patients were moderately depressed, as shown by a mean total score in the HDRS [17] of 20.51 points (SD = 5.10 points; median: 20 points). None of the 100 studied patients proved to have a bipolar disorder. All 100 patients were currently in treatment and received medication and/or psychotherapy. Further demographic and clinical characteristics of the sample are presented in the Tables 2 and 3.

**Table 2:** Demographical characteristics of the sample (*N* = 100)

Variables	Total sample (*N* = 100)	Males (*N* = 39)	Females (N = 61)	*P*
Age in years (Mean (SD))	46.61 (17.07)	46.18 (14.65)	46.89 (18.56)	0.99^a^
Nationality: German (%)	98 (98%)	39 (100%)	59 (96.7%)	0.52^b^
Educational level	–	–	–	0.051^b^
- Pupils (%)	1 (1%)	0 (0%)	1 (1.6%)	–
- Special school leaving certificate (%)	1 (1%)	0 (0%)	1 (1.6%)	–
- Secondary modern school leaving certificate (%)	14 (14%)	1 (2.6%)	13 (21.3%)	–
- Junior high school leaving certificate (%)	38 (38%)	16 (41.0%)	22 (36.1%)	–
- University-entrance diploma (%)	17 (17%)	9 (23.1%)	8 (13.1%)	–
- Technical college / university degree (%)	29 (29%)	13 (33.3%)	16 (26.2%)	–
Partner situation	–	–	–	0.59^b^
- No short-term partner (%)	12 (12%)	7 (17.9%)	5 (8.2%)	–
- No long-term or enduring partner (%)	29 (29%)	12 (30.8%)	17 (27.9%)	–
- Varying partners (%)	3 (3%)	1 (2.6%)	2 (3.3%)	–
- Permanent partner (spouse) (%)	26 (26%)	8 (20.5%)	18 (29.5%)	–
- Permanent partner (no spouse) (%)	30 (30%)	11 (28.2%)	19 (31.1%)	–
Profession	–	–	–	0.35^b^
- Pupil, student, vocational training (%)	9 (9%)	4 (10.3%)	5 (8.2%)	–
- Employed (full-time) (%)	36 (36%)	17 (43.6%)	19 (31.1%)	–
- Employed (part-time) (%)	8 (8%)	1 (2.6%)	7 (11.5%)	–
- Unemployed (%)	19 (19%)	8 (20.5%)	11 (18.0%)	–
- Vocational disability benefit (%)	7 (7%)	3 (7.7%)	4 (6.6%)	–
- Early retirement pension, retirement pension (%)	14 (14%)	6 (15.4%)	8 (13.1%)	–
- Widow’s pension (%)	5 (5%)	0 (0%)	5 (8.2%)	–
- Without employment, not otherwise specified (%)	2 (2%)	0 (0%)	2 (3.3%)	–

Notes: *SD* standard deviation

^a^Mann-Whitney U test; ^b^ Fisher’s exact test (if at least one cell had an expected frequency < 5). All statistical tests had been two-sided

**Table 3:** Clinical characteristics of the sample (N = 100)

Variables	Total sample (*N* = 100)	Males (*N* = 39)	Females (*N* = 61)	*P*
HDRS total score (mean (SD))^d^	20.51 (5.10)	21.92 (4.90)	19.61 (5.05)	0.03^b^
Number of previous depressive episodes (mean (SD))	4.10 (6.88) (*n* = 94)	4.08 (5.52) (n = 37)	4.11 (7.68) (*n* = 57)	0.73^b^
Main diagnosis (according to ICD-10 criteria)	–	–	–	0.61^c^
- F32: Depressive episode (%)^e^	43 (43%)	18 (46.2%)	25 (41.0%)	–
- F33: Recurrent depressive disorder (%)	57 (57%)	21 (53.8%)	36 (59.0%)	–
Duration of the current depressive episode (in months) (Mean (SD))	4.52 (4.92)	3.79 (4.89)	4.98 (4.92)	0.11^b^
Age at the onset of depression (in years) (Mean (SD))	36.11 (18.59)	36.59 (18.27)	35.80 (18.94)	0.66^b^
Number of previous suicide attempts (Mean (SD))	0.53 (1.25)	0.72 (1.38)	0.41 (1.16)	0.02^b^

Notes: HDRS: 17-item version of the Hamilton Rating Scale for Depression [17]; ICD-10: International statistical classification of diseases and related health problems; 10th revision [15]; SD: standard deviation

^a^Fisher’s exact test (if at least one cell had an expected frequency < 5); ^b^ Mann-Whitney U test; ^c^ Chi-square test for two-by-two tables. All statistical tests had been two-sided

^d^There had been two missing values regarding HDRS items 12 and 17 which had been imputed by using the item-specific arithmetical means. Thus, it was possible to compute HDRS sum scores for the total sample (*N* = 100)

^e^All patients in this group were characterized by a first onset of depression

Overall, there were no significant gender differences regarding most of the demographic and clinical variables (*p* ≥ 0.051). However, male patients were found to be significantly more depressed than female patients, as indicated by their HDRS sum scores (21.92 versus 19.61 points; Z = − 2.15; *p* = 0.03) and to have a significantly higher mean number of previous suicide attempts (Z = − 2.24; *p* = 0.02).

### Acute critical life events

96 (96%) patients had at least one acute critical life event within six months prior to the first symptoms of the current depressive episode. Overall, patients had on average 4.36 (SD = 2.98) acute critical life events within this period. Characteristics of acute critical life events in the total sample and in four subsamples (male and female patients; patients with depressive episodes, patients with recurrent depressive disorders) are summarized in Table 4.

**Table 4:** Acute critical life events in the sample (N = 100)

Variables (MEAN (SD))	Total sample (*N* = 100)	Males (*N* = 39)	Females (*N* = 61)	*P*	Patients with depressive episodes (*N* = 43)	Patients with recurrent depressive disorders (*N* = 57)	*P*
Total number of acute critical life events	4.36 (2.98)	4.95 (3.40)	3.98 (2.64)	0.16^a^	4.58 (3.19)	4.19 2.83)	0.60^a^
Total number of foreseeable acute critical life events	2.07 (1.99)	2.41 (2.20)	1.85 (1.82)	0.20^a^	2.19 (2.30)	1.98 (1.73)	0.95^a^
Total number of unforeseeable acute critical life events	2.29 (1.99)	2.54 (2.25)	2.13 (1.81)	0.48^a^	2.40 (2.05)	2.21 (1.96)	0.70^a^
Total number of acute positive critical life events	1.44 (1.71)	1.92 (1.77)	1.13 (1.62)	0.02^a^	1.49 (1.78)	1.40 (1.68)	0.79^a^
Total number of acute neutral critical life events	0.43 (0.70)	0.31 (0.57)	0.51 (0.77)	0.22^a^	0.44 (0.70)	0.42 (0.71)	0.87^a^
Total number of acute negative critical life events	2.49 (1.93)	2.72 (2.33)	2.34 (1.62)	0.75^a^	2.65 (2.07)	2.37 (1.82)	0.52^a^
Total number of acute major critical life events	2.79 (2.01)	3.00 (2.33)	2.66 (1.78)	0.61^a^	3.00 (2.13)	2.63 (1.92)	0.44^a^
Total number of acute minor critical life events	1.57 (1.68)	1.95 (2.01)	1.33 (1.40)	0.15^a^	1.58 (1.83)	1.56 (1.58)	0.90^a^
Mean burden of acute critical life events	3.31 (0.85) (*n* = 96)	3.18 (0.98) (*n* = 37)	3.39 (0.76) (*n* = 59)	0.25^b^	3.23 (0.74) (*n* = 41)	3.37 (0.93) (*n* = 55)	0.45^b^
Mean unpleasantness of acute critical life events	3.50 (0.89) (*n* = 96)	3.31 (0.99) (*n* = 37)	3.61 (0.81) (*n* = 59)	0.10^a^	3.49 (0.82) (*n* = 41)	3.51 (0.95) (*n* = 55)	0.77^a^

Notes: *SD* standard deviation

^a^Mann-Whitney U test; ^b^ t test for independent sample comparison

No group difference was significant at the alpha-adjusted significance level (α = 0.005)

Male patients were found to have significantly more positive acute critical life events within six months prior to the first symptoms of the current depressive episode as compared to female patients (*p* = 0.02; see also Table 4).

Post-hoc analyses revealed that higher HDRS sum scores significantly went along with a higher burden of acute critical life events (rho = 0.25; *p* = 0.01).

In male patients, higher HDRS sum scores were significantly related to a more pronounced unpleasantness of these events (rho = 0.36; *p* = 0.03; *n* = 37).

A significant correlation between mean burden of acute critical life events and HDRS sum scores was found in females (rho = 0.29; *p* = 0.03).

However, all these correlations failed to be significant after controlling for multiple testing.

Differences between patients with depressive episodes and patients with recurrent depressive disorders regarding acute critical life event variables failed to be statistically significant (see Table 4).

### Speed of onset for the current depressive episode

**Fig. 1:**
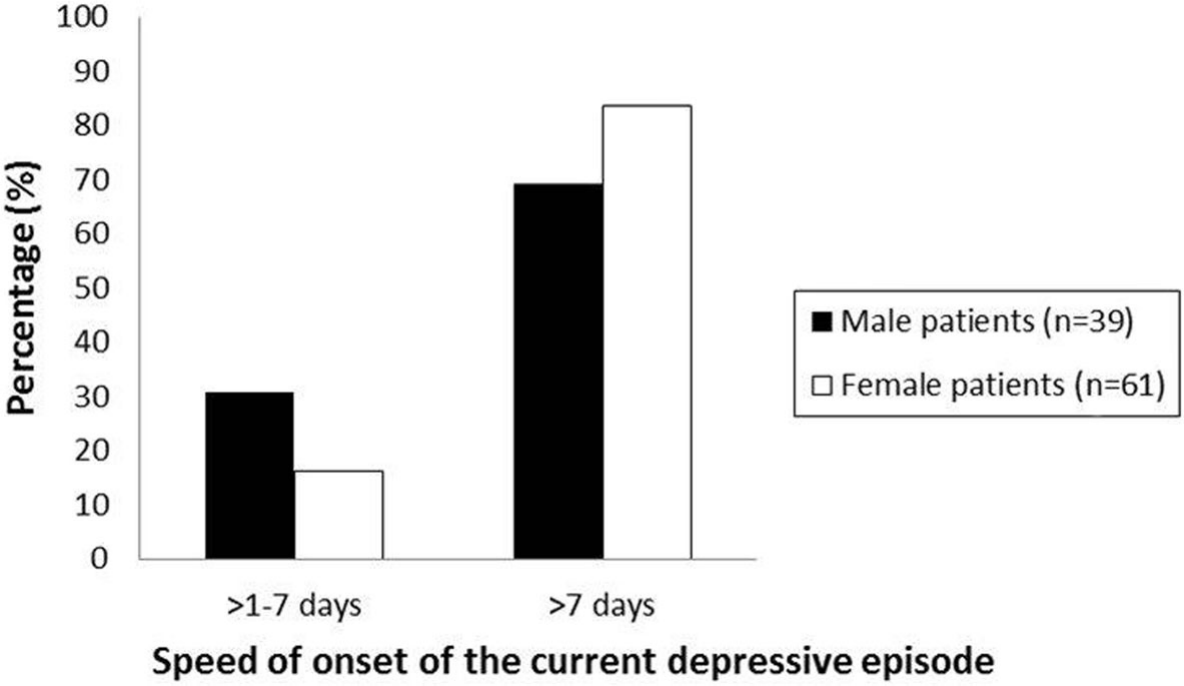
Percentage distribution of the variable “Speed of onset of the current depressive episode” according to estimations of 100 patients suffering from unipolar depressive disorders, for males (*n* = 39) and females (*n* = 61) separately

The mean speed of onset of the current depressive episode in days was 75.46 days (SD = 91.33 days). 22 patients (22%) had a fast onset of the current depressive episode (≤ 7 days), 78 patients (78%) a slow one (> 7 days). Figure 1 demonstrates the frequency distribution for gender-specific speed of onset of the current depressive episode.

Females had a slower onset of the current depressive disorder than males by trend (males: 49.75 days; SD = 51.58 days; females: 91.89 days; SD = 106.65 days; Z = − 1.90; *p* = 0.06). There were no significant associations of age and severity of depression with the speed of onset of the current depressive episode (*p* ≥ 0.45). The same was true for the male subgroup and the female one.

Differences between patients with a depressive episode (ICD-10: F32.x) and patients with recurrent depressive disorders (ICD-10: F33.x) regarding the speed of onset for the current depressive episode (mean ± S.D. (F32.x): 90.25 ± 110.80 days; mean ± S.D. (F33.x): 64.30 ± 72.42 days) were not significant (Z = − 1.09; *p* = 0.28).

### Associations between acute critical life events and speed of onset for the current depressive episode

The correlations between the number, mean burden and unpleasantness of different types of acute critical life events and speed of onset of the current depressive episode are summarized in Table 5.

**Table 5:** Acute critical life events and speed of onset of the current depressive episode in the sample (*N* = 100)

Variables	Total Sample (*N* = 100) R(P)
Total number of acute critical life events	− 0.08^b^ (*p* = 0.42)
Total number of foreseeable acute critical life events	−0.14^b^ (*p* = 0.16)
Total number of unforeseeable acute critical life events	0.002^c^ (*p* = 0.99)
Total number of acute positive critical life events	−0.24^b^ (*p* = 0.02)
Total number of acute neutral critical life events	−0.10^c^ (*p* = 0.33)
Total number of acute negative critical life events	0.13^b^ (*p* = 0.21)
Total number of acute major critical life events	0.10^b^ (*p* = 0.34)
Total number of acute minor critical life events	−0.28^b^ (*p* = 0.005*)
Mean burden of acute critical life events	0.31^a^ (*p* = 0.002*) (*n* = 96)
Mean unpleasantness of acute critical life events	0.33^b^ (*p* = 0.001*) (*n* = 96)

^a^Semi-partial correlation coefficient due to a significant association between a variable regarding critical life events, age and the total score of the Hamilton Depression Rating Scale (17-item version) [17]; ^b^ Semi-partial correlation coefficient due to a significant association between a variable regarding critical life events and age; ^c^ Spearman-Brown correlation coefficient (in cases of non-normal distribution as assessed by the Kolmogorov-Smirnov test)

*p ≤ 0.005 (significant at the alpha-adjusted significance level)

For the total sample, significant negative correlations between the number of acute positive and minor critical life events and speed of onset of the current depressive episode were found (*p* ≤ 0.02). However, only the association between the number of minor acute life events and speed of onset for the current depressive episode remained statistically significant after Bonferroni correction for multiple testing. In line with these findings, lower burden and unpleasantness of acute critical life events were significantly associated with faster onset of the current depressive episode (*p* ≤ 0.002).

These results could be partly confirmed by a linear regression analysis (see Table 6): Slower onset of the current depressive episode was found to be significantly associated with a lower number of acute minor critical life events (β = − 0.23; *p* = 0.02). The final regression model was statistically significant at the 5% level (*p* = 0.02) and explained 4.3% of the variance regarding the speed of onset of the current depressive episode. Neither the number of acute major life events nor the corresponding number of neutral events retained in the regression model (see Table 6).

**Table 6:** Critical life event predictors of the speed of onset for the current depressive episode as derived from a multiple linear regression analysis

Predictor	β	95% CI for β	Standardized β	Standard error	*p* value
Model 1: F = 2.73; df = 3,96; *p* = 0.048; corrected R^2^ = 0.050					
Total number of acute minor critical life events	−15.20	−26.65 to −3.76	−0.28	5.77	0.010
Total number of acute neutral critical life events	−0.22	− 26.93 to 26.49	− 0.002	13.46	0.99
Total number of acute major critical life events	7.71	−1.63 to 17.05	0.17	4.70	0.10
Model 2: F = 4.14; df = 2,97; *p* = 0.019; corrected R^2^ = 0.060					
Total number of acute minor critical life events	−15.23	−26.23 to − 4.22	−0.28	5.55	0.007
Total number of acute major critical life events	7.70	−1.53 to 16.94	0.17	4.65	0.10
Final Model: F = 5.45; df = 1,98; *p* = 0.022; corrected R^2^ = 0.043					
Total number of acute minor critical life events	−12.45	−23.04 to − 1.87	−0.23	5.33	0.022

Notes: The regression model was a linear one with backward elimination (cut-off value: *p* < 0.10). The dependent variable was the speed of onset of the current depressive episode (in days)

β: beta regression coefficient estimates with corresponding 95% confidence intervals (CI) and *p* values; R^2^: the proportion of variance accounted for by the regression model as a whole

### Moderator effects by gender

The interaction effect between the number of acute major life events and gender on the speed of onset for the current depressive episode failed to be significant (F_change_ = 0.145; df = 1,96; *p* = 0.705) (see Table 7). The interaction effect between the number of acute minor life events and gender on this variable was also not significant (F_change_ = 0.782; df = 1,96; *p* = 0.379). The same was true for the interaction effect between the number of acute neutral life events and gender on the speed of onset for the current depressive episode (F_change_ = 0.007; df = 1,96; *p* = 0.931).

**Table 7:** Associations between independent types of acute critical life events and the speed of onset for the current depressive episode: Interaction effects with gender and presence of prior depressive episodes

Step	Added variables	R^2^	R^2^_CHANGE_	F_CHANGE(DF)_	P_CHANGE_
Interaction effects with gender (males/females)					
Model 1: Major acute life events and gender					
1	Major acute life events	0.007	0.007	0.705 (1,98)	0.403
2	Gender	0.062	0.055	5.668 (1,97)	0.019
3	Gender x Major acute life events	0.063	0.001	0.145 (1,96)	0.705
Model 2: Minor acute life events and gender					
1	Minor acute life events	0.053	0.053	5.450 (1,98)	0.022
2	Gender	0.088	0.035	3.750 (1,97)	0.056
3	Gender x Minor acute life events	0.095	0.007	0.782 (1,96)	0.379
Model 3: Neutral acute life events and gender					
1	Neutral acute life events	0.003	0.003	0.274 (1,98)	0.602
2	Gender	0.058	0.056	5.735 (1,97)	0.019
3	Gender x Neutral acute life events	0.059	0.00007	0.007 (1,96)	0.931
Interaction effects with the presence of prior depressive episodes (PPDE) (yes/no)					
Model 1: Major acute life events and PPDE					
1	Major acute life events	0.007	0.007	0.705 (1,98)	0.403
2	PPDE	0.025	0.018	1.791 (1,97)	0.184
3	PPDE x Major acute life events	0.029	0.004	0.417 (1,96)	0.520
Model 2: Minor acute life events and PPDE					
1	Minor acute life events	0.053	0.053	5.450 (1,98)	0.022
2	PPDE	0.073	0.020	2.131 (1,97)	0.148
3	PPDE x Minor acute life events	0.073	0.00003	0.003 (1,96)	0.956
Model 3: Neutral acute life events and PPDE					
1	Neutral acute life events	0.003	0.003	0.274 (1,98)	0.602
2	PPDE	0.023	0.020	2.006 (1,97)	0.160
3	PPDE x Neutral acute life events	0.037	0.014	1.406 (1,96)	0,239

Notes: R^2^ = proportion of explained variance; df = degree of freedom

In all regression analyses, the criterion was the speed of onset for the current depressive episode (in days)

No result was significant at the alpha-adjusted significance level (α = 0.017)

### Moderator effects by the presence of prior depressive episodes

The interaction effect between the number of acute major life events and the presence of prior depressive episodes (yes (ICD-10: F33.x) versus no (ICD-10: F32.x)) on the speed of onset for the current depressive episode did not reach statistical significance (F_change_ = 0.417; df = 1,96; *p* = 0.520) (see Table 7). The interaction effect between the number of acute minor life events and presence of prior depressive episodes on this variable was not significant, too (F_change_ = 0.003; df = 1,96; *p* = 0.956). The same was true for the interaction effect between the number of acute neutral life events and presence of prior depressive episodes on the speed of onset for the current depressive episode (F_change_ = 1.406; df = 1,96; *p* = 0.239).

## Discussion

This is the first study that examined differences in acute critical life events prior the occurring of first symptoms of depression in the context of the speed of onset of the depression. The majority of the enrolled depressed patients (96%) irrespective of the speed of onset of their depression reported at least one acute critical life event within six months before the onset of first symptoms of their depression. Past studies have shown a relationship between the onset of depressive disorders and the occurring of critical life events prior the beginning of the depression [1]. Thus, our results are consistent with previous studies.

Overall, patients with a fast onset of the depression experienced more often acute minor and positive critical life events in the 6 months prior the beginning of first symptoms of their depression. Correspondingly, lower burden and unpleasantness of acute critical life events were significantly associated with faster onset of the current depressive episode. The number of acute minor critical life events in the 6-month period preceding the onset of first symptoms of the current depressive episode could be identified as the life event variable with the most pronounced impact on the onset of a depressive episode. Studies have shown that genetic factors influence the risk of onset of major depression by altering sensitivity of individuals to the cumulative depression-inducing effect of multiple stressful life events [19, 20]. The association of a fast beginning of the depression and acute minor critical life events prior the beginning is perhaps an indication for a stronger genetic diathesis in patients with a fast beginning of their depression. However, we could not show that individuals were more sensitive to the effects of acute minor life events after they had a depressive episode such that acute minor life events were more likely to predict recurrence of depression than the first onset of a depressive episode [8] since our moderator analyses revealed that, in the presence of prior depressive episodes, acute minor life events were not more closely associated with the speed of onset for the current depressive episode than in patients without such a history. However, we cannot exclude that having a prior episode of depression had a significant influence on the association between critical life events and speed of onset for the current depressive episode if the accumulation of minor life events in a longer period before the onset of the current depressive episode (more than six months) was considered. Thus, it would be interesting to investigate these associations in the context of a study focusing on a longer period preceding the onset of depressive episodes.

For other types of acute life events (major and neutral ones), the presence of prior depressive episodes failed to be a moderator for the association between critical acute life events and the speed of onset for the current depressive episodes, too. Therefore, having a first onset or prior episode of depression does not seem to have a significant general influence on the association between acute critical life events and speed of onset of the current depressive episode.

Regarding gender differences for the occurring of acute critical life events prior the onset of first symptoms of the depression they failed to be significant after correcting for multiple testing while male patients experienced significantly more often positive acute life events than female patients at the 5% level. Past studies with respect to the total number of life events prior the beginning of the depression showed partially no gender differences. This is in contrast with other studies which indicated that women are more often exposed to negative life events than men [3].

While females had a slower onset of the current depressive disorder than males by trend the association between different types of acute life events (major, minor, neutral) and the speed of onset for the current depressive episode was not moderated by gender. Thus, we have to reject our hypothesis that acute critical life events trigger a fast onset of depressive episodes more often in women than in men.

Our findings with respect to the speed of onset of depression are in accordance with previous studies: Similar to our previous findings also in this sample there was no association between the speed of onset and the severity of the depression [11].

Overall, the associations between the number of acute life events and speed of onset for the current depressive episode failed to be statistically significant after correcting for multiple testing (with the exception of minor acute life events) and were only low to middle. These findings support the assumption, that a fast onset of the depression is not strongly influenced by external critical life events and that speed of onset is an individual clinical characteristic of depressive episodes which is likely to point to underlying neurobiological differences.

Moreover, higher burden and unpleasantness of acute critical life events were significantly associated with slower onset of the current depressive episode: Regarding patients with slower onset of the current depressive episode, they tend to estimate acute critical life events before the onset of first symptoms of their current depressive episode to be more unpleasant than patients with fast onset of the depressive episode (within one week). This finding possibly reflects a generally increased sensitivity in this subgroup of depressed patients regarding acute critical life events. So, it can be speculated that fewer critical life events can trigger a depressive episode in this group as compared to patients with a fast onset of depression if the unpleasantness of the critical life events exceeds a critical cut-off value. However, it must be emphasized that the corresponding correlation coefficients (0.31 and 0.33, respectively) were only middle and should therefore be interpreted with caution.

One strength of this study is the systematical investigation of the association between critical life events and the speed of onset of depression in a quite large clinical sample by using well-established instruments (MEL, ODI).

Limitations of this study include the lack of control of the possible influence of pharmacotherapy and psychotherapy on the speed of onset of depression as well as the not blinded design of the study. The two possible pathways to a fast onset of depressive episodes (i.e., provoking event and depression onset, bipolarity liability and depression onset) did not allow for a very precise specification of the hypotheses. Moreover, we decided to investigate inpatients with depressive disorders because we expected more frequent and more pronounced acute critical life events (due to the higher severity of depression) in this group as compared to outpatients suffering from depression. Thus, our findings cannot be readily transferred to outpatients with depressive disorders. In addition, all raters measured all three types of information (HDRS, ODI, MEL). Thus we cannot exclude the possibility that the information of the ODI had an impact on the MEL interview or vice versa. Unfortunately, we could not provide reliability statistics for clinical diagnoses because independent diagnostic procedures had not been performed. A further problem is associated with the fact that the ODI does not explicitly cover chronic or residual symptoms of depression. Thus, it cannot be ensured that the participant had fully recovered from any prior depressive episode. Many patients will delay coming in until months after a depression has started, and those in regular treatment may not always go to see their doctors on a regular basis. By then, the patients may suffer from recall bias which could have influenced the patients’ remarks about speed of onset of their current depressive episodes in our sample. The exclusion of patients with acute suicidality had been performed in view of difficulties to include them in a clinical study and can thus be justified. Nevertheless, this exclusion has to be regarded as problematic since it may have had a significant impact on the speed of depression onset following life events and generalizability to other inpatient populations with unipolar depressive disorders as well.

Another point refers to the fact that we did not focus our analyses on the association of major critical life events and speed of onset of depressive episodes. One might argue that this might have diluted the information value of severe events with the potentially irrelevant information value of the non-severe life stress. However, minor critical life events can trigger depressive episodes in individuals with a depressive disposition. Thus we abstained from restricting the analyses to major critical life events. This would have not been an adequate approach in view of the complexity of the relationships between critical life events and the speed of onset of depressive episodes.

For future studies, it would be interesting to investigate the role of genetic factors as potential moderators of the association between speed of onset of depressive episodes and acute critical life events. Moreover, it would be interesting to focus on severe critical life events occurring within 3 months or less of depression onset in the context of a future study since there are findings suggesting that the majority of severe critical life events provoking depression occur within this timeframe [21]. Last, but not least it has to be emphasized that it will be very important to replicate our significant findings in the context of a larger independent study.

## Conclusions

Fast onset of a depressive episode was not significantly associated with more acute critical life events in the 6 months before the onset of the depression. Moreover, the association between the number of different types of acute critical life events in the half-year period preceding the onset of unipolar depressive disorders and speed of onset for the current depressive episode is neither dependent from gender nor the presence of prior depressive episodes. These findings support the assumption that speed of onset of depression is not strongly influenced by external triggers, e.g. acute critical life events.

## Additional file

Additional file 1: **Table S1.** Inter-correlations between different variables reflecting acute critical life events derived from the Munich Interview for the Assessment of Life Events and Conditions (MEL). (DOCX 18 kb)
