# Supplementary material for: Association between acute critical life events and the speed of onset of depressive episodes in male and female depressed patients
Source: BMC Psychiatry. 2018 Oct 16;18:332. doi: 10.1186/s12888-018-1923-4 (PMC6192272; doi:10.1186/s12888-018-1923-4)
Supplement: Supplementary file 1 — Table S1. Inter-correlations between different variables reflecting acute critical life events derived from the Munich Interview for the Assessment of Life Events and Conditions (MEL). (DOCX 18 kb) [file 12888_2018_1923_MOESM1_ESM.docx]

**Supplemental Table 1: Inter-correlations between different variables reflecting acute critical life events derived from the Munich Interview for the Assessment of Life Events and Conditions (MEL)**

| **Variables** | **Total number of critical life events** | **Total number of acute critical life events** | **Total number of unforeseeable acute critical life events** | **Total number of foreseeable acute critical life events** | **Total number of acute positive critical life events** | **Total number of acute neutral critical life events** | **Total number of acute negative critical life events** | **Total number of acute minor critical life events** | **Total number of acute major critical life events** | **Mean unpleasantness of acute critical life events** | **Mean burden of acute critical life events** |
| --- | --- | --- | --- | --- | --- | --- | --- | --- | --- | --- | --- |
| **Total number of critical life events** | 1.00 | 0.85*** (n=100) | 0.70*** (n=100) | 0.52*** (n=100) | 0.55*** (n=100) | 0.17 (n=100) | 0.75*** (n=100) | 0.54*** (n=100) | 0.80*** (n=100) | -0.11 (n=96) | -0.17 (n=96) |
| **Total number of acute critical life events** | --- | 1.00 | 0.77*** (n=100) | 0.68*** (n=100) | 0.70*** (n=100) | 0.25* (n=100) | 0.77*** (n=100) | 0.70*** (n=100) | 0.85*** (n=100) | -0.33*** (n=96) | -0.38*** (n=96) |
| **Total number of unforeseeable acute critical life events** | --- | --- | 1.00 | 0.11 (n=100) | 0.41*** (n=100) | 0.19 (n=100) | 0.73*** (n=100) | 0.43*** (n=100) | 0.74*** (n=100) | -0.04 (n=96) | -0.12 (n=96) |
| **Total number of foreseeable acute critical life events** | --- | --- | --- | 1.00 | 0.64*** (n=100) | 0.19 (n=100) | 0.38*** (n=100) | 0.61*** (n=100) | 0.50*** (n=100) | -0.45*** (n=96) | -0.44*** (n=96) |
| **Total number of acute positive critical life events** | --- | --- | --- | --- | 1.00 | 0.03 (n=100) | 0.21* (n=100) | 0.80*** (n=100) | 0.38*** (n=100) | -0.78*** (n=96) | -0.63*** (n=96) |
| **Total number of acute neutral critical life events** | --- | --- | --- | --- | --- | 1.00 | 0.03 (n=100) | 0.33*** (n=100) | 0.16 (n=100) | -0.18 (n=96) | -0.24* (n=96) |
| **Total number of acute negative critical life events** | --- | --- | --- | --- | --- | --- | 1.00 | 0.23* (n=100) | 0.90*** (n=100) | 0.30** (n=96) | 0.13 (n=96) |
| **Total number of acute minor critical life events** | --- | --- | --- | --- | --- | --- | --- | 1.00 | 0.27** (n=100) | -0.70*** (n=96) | -0.80*** (n=96) |
| **Total number of acute major critical life events** | --- | --- | --- | --- | --- | --- | --- | --- | 1.00 | 0.08 (n=96) | 0.09 (n=96) |
| **Mean unpleasantness of acute critical life events** | --- | --- | --- | --- | --- | --- | --- | --- | --- | 1.00 | 0.80*** (n=96) |
| **Mean burden of acute critical life events** | --- | --- | --- | --- | --- | --- | --- | --- | --- | --- | 1.00 |

**Notes:** The numbers in the cells of the table refer to Spearman-Brown correlation coefficients. * p ≤ 0.05; ** p ≤ 0.01; *** p ≤ 0.001.
